# What is your diagnosis?

**DOI:** 10.4274/jtgga.galenos.2020.2019.0208

**Published:** 2021-02-24

**Authors:** Gülşah Aynaoğlu Yıldız, Sevilay Özmen, Ömer Erkan Yapça, Emsal Pınar Topdağı Yılmaz, Ragıp Atakan Al

**Affiliations:** 1 Clinic of Obstetrics and Gynecology, Nenehatun Maternity Hospital, Erzurum, Turkey; 2 Department of Pathology, Atatürk University Faculty of Medicine, Erzurum, Turkey; 3 Department of Obstetrics and Gynecology, Atatürk University Faculty of Medicine, Erzurum, Turkey

A 29-year-old, 20-month pregnant woman was admitted to our center, since she had a history of hydrocephalus and anencephaly in her previous pregnancy. There was no history of taking folic acid tablets before pregnancy, but she took them in the first trimester of her current pregnancy. A detailed obstetric history was taken and it was determined that the patient had a consanguineous marriage with a second cousin. She had a history of three previous pregnancies. Two of them resulted in the birth of healthy babies and one was terminated because of hydrocephalus and anencephaly.

Fetal ultrasonographic findings were: a hyperechogenic, cystic structure, measuring 28x31 mm in the right lobe of the liver, hyperechogenic and polycystic left kidney, cystic hygroma, pulmonary hypoplasia and single umblical artery. On magnetic resonance imaging, there were no cerebellar vermis and an absent corpus callosum (Figure 1). Informed consent was obtained and amniocentesis was performed. The patient decided to terminate the pregnancy without waiting for the result of amniocentesis, so the pregnancy was terminated and the fetus was sent for an autopsy examination.

The male fetus weighed 476 g. The crown rump length, chest circumference and abdominal circumference were measured as 18 cm, 19 cm, 21 cm, respectively. It was seen that cerebellar vermis and corpus callosum were absent. The fetus had bulging eyes, broad nose, depressed nasal bridge, folded ears, large mouth with a protruding tongue, long philtrum, and small chin. An increase in his nuchal fold thickness was found with the help of an external examination (Figure 2).

On dissection and internal examination: cardiac defect and diaphragmatic hernia were not observed. A serous cystic structure with a size of 28x31 mm was observed in the right lobe of the liver (Figure 3). The left kidney parenchyma could not be observed. Pulmonary hypoplasia was observed (total lung weight was 21 grams). Amniocentesis reported a normal karyotype.

## Answer

Fryns syndrome is a rare, autosomal recessive disorder with multiple congenital anomalies and has a prevalence of about 0.7 per 10,000 births (1). It is characterized by diaphragmatic defects, typical face, distal digital or nail hypoplasia, pulmonary hypoplasia and some associated anomalies which may include polyhydramnios, cloudy corneas and/or microphthalmia, orofacial clefting, renal dysplasia/renal cortical cysts, and/or malformations including the cardiovascular system, gastrointestinal system, brain or the genitals. It is also closely related with consanguineous marriage. The diagnosis is based on clinical findings and is made with the presence of three criteria (2,3). Regarding genetic analysis, fetal karyotypes of cases are usually normal, so it is important to diagnose the syndrome at autopsy. Fryns syndrome, known as the most common autosomal recessive disorder causing congenital diaphragmatic hernia (CDH), is responsible for 4-10% of all patients with CDH. Diaphragmatic defects are found in almost all cases with Fryns syndrome (4). A limited number of cases with Fryns syndrome without diaphragmatic hernia have been identified in the literature (5).

The purpose of this autopsy case report is to show that Fryns syndrome can be diagnosed without CDH and to show its association with rare abnormalities, such as cystic hygroma, agenesis of the corpus callosum and the cerebellar vermis.

Fryns syndrome is one of the most common syndromes associated with CDH and is reported in up to 10% of patients with CDH. Despite the fact that there wasn’t any diaphragmatic defect, other findings of our patient were similar to the rest of the typical diagnostic findings, with the exception of cystic hygroma, and some other defects not previously described. Several chromosomal abnormalities show symptoms similar to Fryns syndrome. Therefore, the diagnosis of Fryns syndrome can only be made if the karyotype is normal and the diagnosis is confirmed by autopsy. In this case, autopsy findings were very useful in making a differential diagnosis. Based on the ultrasonographic findings, the first idea was chromosomal anomaly. However, amniocentesis was reported as normal. We did not recommend non-invasive prenatal testing as an alternative to amniocentesis as non-invasive prenatal testing is not suitable for genetic evaluation of ultrasound anomalies (6). Simpson-Golabi-Behmel syndrome, an X-linked overgrowth syndrome resulting from deletions or mutations in the *GPC3* gene, and conditions with hypoplasia or absence of the distal phalanges such as DOOR syndrome (deafness, onychodystrophy, osteodystrophy, and mental retardation), Schinzel-Giedion syndrome, and Rudiger syndrome should be considered in differential diagnosis. However, heterozygous de novo mutation in the *SETBP1* gene is characteristic of Schinzel-Giedion syndrome and none of the patients with Rudiger syndrome has diaphragmatic hernia. The diagnosis of Fryns syndrome without diaphragmatic hernia is quite difficult. Although this patient is diagnosed as Fryns syndrome, it cannot clearly be differentiated from Rudiger syndrome and Schinzel-Giedion syndrome. Therefore, these diseases can be called Fryns-like syndromes (7). In the presence of cystic hygroma, other signs of Fryns syndrome should be carefully monitored, since the risk of recurrence is 25 percent per pregnancy and should be kept in mind during subsequent pregnancies.

## Figures and Tables

**Figure 1:**
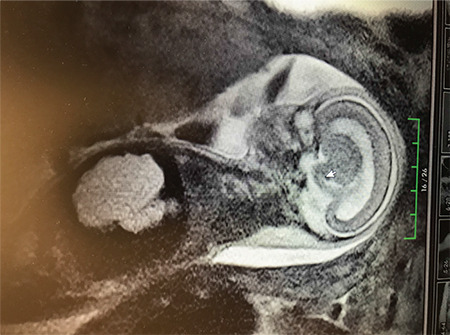
Magnetic resonance imaging of agenesis of corpus callosum

**Figure 2:**
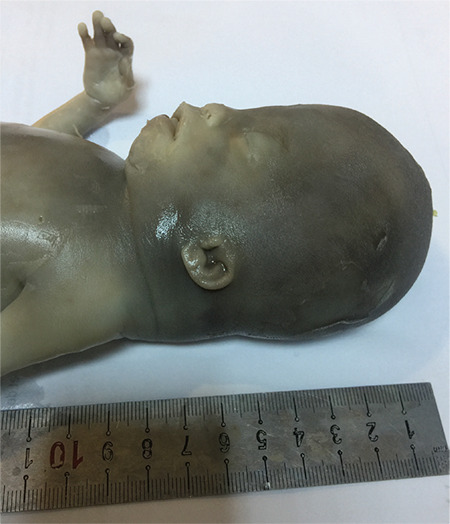
Bulging eyes, broad nose, depressed nasal bridge, folded ears, large mouth with a protruding tongue, long philtrum, small chin and increase in nuchal fold thickness

**Figure 3:**
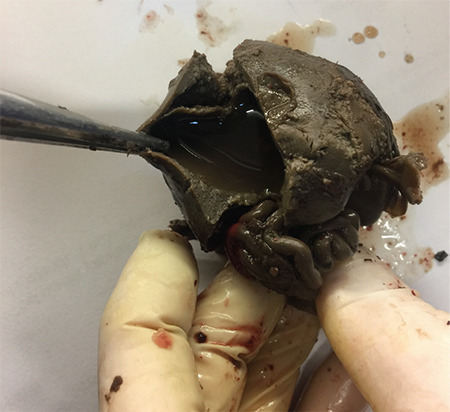
Hyperechogenic cystic structure measuring 28x31 mm in the right lobe of the liver

## References

[1] Nirmaladevi M, Kurup S, Ajitha EV (2012). Fryns syndrome in monozygotic twins: a case report with review of literature. Int J Morphol.

[2] Lin AE, Pober BR, Mullen MP, Slavotínek AM (2005). Cardiovascular malformations in fryns syndrome: is there apathogenic role for neural crest cells?. Am J Med Genel A.

[3] Slavotinek A (2007.). Fryns syndrome. GeneReviews® [Internet].

[4] Dillon E, Renwick M (1993). Antenatal detection of congenital diaphragmatic hernias: the Northern region experience. Clin Radiol.

[5] Alessandri L, Brayer C, Attali T, Samperiz S, Tiran-Rajaofera I, Ramful D, et al (2005). Fryns syndrome without diaphragmatic hernia. Report on a new case and review of the literature. Genet Couns.

[6] Beulen L, Faas BHW, Feenstra I, Van Vugt JMG, Bekker MN (2017). Clinical utility of non-invasive prenatal testing in pregnancies with ultrasound anomalies. Ultrasound Obstet Gynecol.

[7] Alessandri JL, Cuillier F, Malan V, Brayer C, Grondard M, Jacquemot- Dekkak L, et al (2014). Fryns syndrome without diaphragmatic hernia, DOOR Syndrome or fryns-like syndrome? Report on patients from Indian Ocean Islands. Am J Med Genet A.

